# Differences in the Relative External Load Demands of Pre-Competitive Warm-Ups and Official Matches in Semi-Professional Football Players: A Pilot Study Considering Specific Positions

**DOI:** 10.3390/jfmk10020182

**Published:** 2025-05-17

**Authors:** José María Escudero-Ferrer, Luis Manuel Martínez-Aranda, Manuel Sanz-Matesanz, Konstantinos Spyrou, Pedro E. Alcaraz, Javier Raya-González

**Affiliations:** 1 Facultad de Deporte, UCAM Universidad Católica de Murcia, 30107 Murcia, Spain; jmescudero@ucam.edu (J.M.E.-F.); kspyrou@ucam.edu (K.S.); palcaraz@ucam.edu (P.E.A.); 2 Department of Sports and Computer Sciences, Faculty of Sports Sciences, Universidad Pablo de Olavide, 41013 Seville, Spain; 3 Science-Based Training Research Group (SEJ-680), Physical Performance and Sports Research Center, Universidad Pablo de Olavide, 41013 Seville, Spain; 4 Faculty of Health Sciences, European University Miguel de Cervantes, 47012 Valladolid, Spain; msanzm@uemc.es; 5 UCAM Research Center for High Performance Sport (CIARD), UCAM Universidad Católica de Murcia, 30107 Murcia, Spain; 6 Strength and Conditioning Society (SCS), 30107 Murcia, Spain; 7 Grupo de Investigación en Deporte y Educación Física para el Desarrollo Personal y Social (GIDEPSO), Department of Specific Didactics, Faculty of Education Sciences and Psychology, University of Córdoba, 14004 Córdoba, Spain; rayagonzalezjavier@gmail.com

**Keywords:** GPS tracking, load monitoring, high-speed running, team sports, match preparation

## Abstract

**Background:** A pre-competition warm-up is considered a key strategy for optimising physical preparedness and potentially reducing injury risks in football. Programmes such as FIFA 11+ have demonstrated efficacy in this regard. Its effectiveness depends on alignment with match demands. This study compares the relative external load demands of warm-ups and matches in semi-professional football players, focusing on positional differences. **Objective**: The goal of this study was to evaluate whether warm-ups adequately prepare players for match demands and to explore positional variations. **Methods:** This is a retrospective study that analysed 19 semi-professional male players during the 2023/2024 season. External load demands (m/min) were measured using a GPS, covering the total distance (TD), speed zones (DZ1–DZ5), accelerations (ACCs), and decelerations (DECs). Paired *t*-tests and effect size calculations compared team-wide and position-specific demands. **Results**: Match demands significantly exceeded warm-up demands across all distance-related variables, except for DZ1 (67.06 vs. 66.40 m/min for warm-ups and games, respectively). The greatest differences were observed in TD (80.73 vs. 107.12 m/min; −26.39%) and DZ2–DZ3 (−17.42 and −4.89%, respectively). A positional analysis revealed that concerning DZ1, midfielders covered more distance during competitions (67.62 vs. 65.04 m/min; −2.58%), while full-backs covered more during the pre-competition warm-up (69.01 vs. 66.86 m/min; 2.14%). Additionally, midfielders, wingers, and forwards experienced higher match demands in DECs (1.04; 1.12, and 1.18 nº/min; range = 0.23–3.13%), whereas central defenders showed higher values during the pre-competition warm-up (1.14 nº/min; 0.13%). No significant differences were found for ACCs across any position; however, central defenders showed higher nº ACCs during warm-up (1.04 vs. 0.97 nº/min). **Conclusions:** These findings enable clubs and coaches to redesign their warm-up protocols to align as closely as possible with the demands of matches, particularly in high-speed zones, to enhance readiness, thereby increasing the effectiveness of warm-ups in football competitions. Additionally, this approach allows for the individualisation of warm-up routines based on the player’s specific position.

## 1. Introduction

A pre-competition warm-up has been widely implemented in team sports as a strategy to enhance performance and contribute to injury prevention. Its effectiveness, however, depends on several factors, including structure, intensity, and individualisation [1]. Structured protocols, such as FIFA 11+, have demonstrated that a structured warm-up programme can prevent injuries in young football players [2].

This is due to its positive effects, particularly on muscle temperature, metabolic reactions, blood flow, nerve conductivity, anaerobic energy supply, oxygen consumption, and the ability to generate force over time [3,4]. However, if applied excessively in terms of volume and intensity, the effects of this strategy could be detrimental, depleting glycogen stores and excessively increasing body temperature, leading to a level of fatigue that hinders subsequent performance [5]. For this reason, an appropriate design of the pre-competition warm-up is essential to maximise its positive effects.

In football, where high-intensity actions, such as jumps, sprints, and changes of direction, are highly prevalent [6] and significantly influence sporting success [7], a pre-competition warm-up is crucial for preparing players to perform these actions optimally. Generally, the structure of a football pre-competition warm-up consists of the following phases: a general cardiovascular phase combined with dynamic stretching; neuromuscular exercises aimed at injury prevention; exercises to enhance post-activation potentiation; specific technical–tactical drills; and short-duration, high-intensity actions [8].

To optimise a pre-competition warm-up, research has examined the effects of modifying certain parameters. For instance, Ben Brahim et al. [9] studied how the structure (i.e., the order of exercises) of a pre-competition warm-up influenced football players’ physical performance. They observed that concluding the warm-up with neuromuscular activities was effective in improving ball speed, agility, and vertical jump capacity. Regarding duration, Taylor et al. [10] concluded that a shorter warm-up was more practical, allowing for the remaining time to be used for tactical preparation before the match. However, van den Tillaar and von Heimburg [11] found that both short and long warm-ups were equally effective for repeated sprint performance. Another variable, warm-up intensity [12], has also been studied. Despite these efforts, no clear consensus has been reached regarding the optimal pre-competition warm-up.

One possible criterion for optimising a pre-competition warm-up is its degree of similarity, in terms of workload demands, to the official match. To date, only two studies have investigated this correspondence [13,14]. Casamichana et al. [14] found that the external load demands recorded during a pre-competition warm-up corresponded to percentages ranging from 2.2% (i.e., sprint distance) to 18.4% (i.e., total distance) of match demands. Similarly, Williams et al. [13] examined how a pre-competition warm-up contributed to the external load demands of official matches in female football players competing in the top tier of U.S. collegiate football. They observed that the implemented warm-up covered between 21.56% (i.e., total distance) and 36.70% (i.e., number of sprints) of match demands.

However, these studies only reported the percentage of the external load covered during the pre-competition warm-up relative to the total match demands. Therefore, it would be beneficial to examine the similarity in terms of density (m/min) between the warm-up and the match. This information would significantly aid in optimising pre-competition warm-up strategies and, consequently, players’ performance.

Given the differences observed in external load demands based on specific playing positions in football [15,16], it seems essential to compare the relative external load demands (m/min) of a pre-competition warm-up and an official match on an individualised basis for each playing position. For instance, García-Calvo et al. [15] reported that high metabolic load distances vary significantly according to competitive levels and playing positions, with midfielders and full-backs typically covering greater distances. Similarly, Lobo-Triviño et al. [16] showed that the frequency and intensity of tactical sprint actions are influenced by the match status and positional roles, reinforcing the importance of tailoring physical preparation to specific positional demands. This need for future research was highlighted by Casamichana et al. [14].

To address these gaps in the literature, external load data were recorded during pre-competition warm-ups and official matches over an entire season. The primary objective of this study was to compare the relative external load demands (m/min) of a pre-competition warm-up and official matches in semi-professional football players. Based on previous studies [13,14], we hypothesised that the relative external load demands (m/min) of the pre-competition warm-up would be significantly lower than those of the match but closely aligned.

Unlike previous studies that reported only the percentage of the absolute load covered during the warm-up [13,14], the present study provides a novel perspective by comparing the relative external load (m/min) between the warm-up and match. This approach offers a more accurate reflection of movement intensity and is particularly relevant for tailoring warm-up strategies. Additionally, to our knowledge, no prior study has conducted a position-specific analysis of relative external loads in this context (warm-up vs. game/match) These two elements—the use of load density and the inclusion of positional roles—constitute the main contribution and novelty of the present research.

## 2. Materials and Methods

### 2.1. Study Design

A comparative, retrospective, and observational design was employed to compare the relative external load demands (m/min) of a pre-competition warm-up and official matches in semi-professional football players. All regular league matches played during the 2023/2024 season were included in this study, with external load demands monitored during both matches and pre-competition warm-ups. Only players who started the match and played for at least 65 min were considered, resulting in 225 observations from 19 footballers. Substitutes and matches with extra time were excluded. Matches were played in official stadiums (FIFA recommendations: ~105 × 68 m) on both natural and artificial grass surfaces. External load demands were tracked using global positioning system (GPS) devices worn by each player.

### 2.2. Participants

Nineteen Spanish football players (age = 27.8 ± 4.8 years; weight = 74.2 ± 4.9 kg; height = 179.8 ± 4.7 cm; body mass index = 22.9 ± 0.7 kg/m^2^), classified as level 3 (i.e., highly trained/national level) according to McKay et al. [17], voluntarily participated in this study. The players were members of the same team, which competed in the 2nd RFEF division during the 2023/2024 season. The team finished eighth in the regular season standings (out of 18 teams). During the experimental period, players participated in five weekly training sessions in addition to an official match on the weekend. All matches included in the analysis were regular season games, not playoff fixtures. This sample size is comparable to that of previous GPS-based studies in football and was considered sufficient for the objectives of this research. Given the within-subject, repeated-measures design (225 observations across 19 players), the statistical power was optimised despite the limited number of individual participants. Moreover, this study was conceived as a pilot exploration, aiming to provide initial evidence on relative load differences and positional variations, rather than to yield generalisable conclusions for all football populations.

All players underwent a comprehensive medical examination to verify their health status before participating in the study, ensuring they were free of illnesses or injuries during the experimental period. Goalkeepers were excluded from the analysis due to their specific role during matches. All participants were informed about the procedures, methods, benefits, objectives, and potential risks involved in the study before providing their written informed consent. The study was conducted in accordance with the Declaration of Helsinki (2013) and approved by the Ethics Committee of the Catholic University of Murcia (code: CE022106; date: 1 March 2021).

### 2.3. Procedures

#### 2.3.1. External Load Variables

To analyse the external load demands of the football players, participants wore VectorS7 GPS devices. These systems, manufactured by Catapult Sports (Catapult Sports, Melbourne, Australia), were positioned in the centre of the upper back using a vest, with each player using the same device throughout the study. The device includes a triaxial accelerometer, a triaxial gyroscope, and a triaxial magnetometer, each with a sampling frequency of 100 Hz, while the GPS has a sampling frequency of 10 Hz. Previous studies have demonstrated that these GPS devices exhibit high reliability [18,19].

Specifically, the monitored external load variables, relative to time (m/min), included the following: the total distance covered by the players (TD), the distance covered at 0 to 12 km·h^−1^ (DZ1), the distance covered at 12.1 to 18 km·h^−1^ (DZ2), the distance covered at 18.1 to 21 km·h^−1^ (DZ3), distance covered at 21.1 to 24 km·h^−1^ (DZ4), the distance covered at speeds exceeding 24 km·h^−1^ (DZ5), the distance covered at speeds greater than 19 km·h^−1^ per minute (TD > 19/min), the distance covered at speeds greater than 25 km·h^−1^ per minute (TD > 25/min), the number of accelerations exceeding 3 m·s^−2^ (ACC), and the number of decelerations exceeding −3 m·s^−2^ (DEC) performed by the football players.

#### 2.3.2. Pre-Competitive Warm-Up

Before each official match, the team performed the same standard (not individualised) pre-competitive warm-up, which was conducted by the same physical trainer during the whole experimental period. This warm-up lasted 20 min, and was divided into 3 blocks: (i.e., mobility and strength, specific game actions, and activation). Table 1 shows the performed warm-up, specifying each of the tasks included.

#### 2.3.3. Statistical Analysis

The results are presented as the mean ± standard deviation (SD). The Shapiro–Wilk test was applied to assess data normality, and Levene’s test was used to evaluate the homogeneity of variance. A paired samples *t*-test was conducted to analyse differences in relative external load demands (m/min) between the pre-competitive warm-up and the official match. Mean differences were calculated using the following formula: mean difference (%) = ((mean 1−mean 2)/mean 2) × 100. Additionally, Cohen’s effect size (ES) [20] was calculated. The following thresholds were used to interpret the ES: trivial (<0.2), small (0.2–0.5), moderate (0.5–0.8), and large (>0.8). Data analysis was performed using the JASP software version 0.16.3.0 (University of Amsterdam, Amsterdam, The Netherlands). Statistical significance was set at *p* < 0.05.

## 3. Results

Table 2 presents the differences in relative external load demands (m/min) between the pre-competitive warm-up and the official match for the entire team. During competition, relative external load demands were higher than during the pre-competitive warm-up across all distance variables (*p* < 0.001; ES = −0.22 to −2.17, small to large), except for DZ1 (*p* = 0.198; ES = 0.22, small).

The differences in relative external load demands (m/min) between the pre-competitive warm-up and the official match for different specific positions are shown in Table 3, Table 4, Table 5, Table 6 and Table 7. Relative external load demands during official matches were higher than during the pre-competitive warm-up across all specific positions for DT, D2, D3, D4, and D5 (*p* < 0.001; ES = −1.36 to −4.60, large). For D1, significant differences were found in favour of competition for midfielders (*p* < 0.033; ES = −0.32, small) and in favour of the pre-competitive warm-up for full-backs (*p* = 0.009; ES = 0.36, small) and wide players (*p* = 0.014; ES = 0.41, small).

Additionally, significant differences were observed in favour of competition for decelerations (DECs) among midfielders, wide players, and forwards (*p* < 0.001; ES = −0.70 to −1.22, moderate to large), while significant differences were found in favour of the pre-competitive warm-up for central defenders (*p* < 0.001; ES = 0.58, moderate), with no significant differences observed for full-backs (*p* = 0.180; ES = −0.18, small).

Finally, no significant differences were found for accelerations (ACCs) across any specific position.

## 4. Discussion

The primary aim of this study was to compare the relative external load demands (m/min) of the pre-competition warm-up and official matches in semi-professional football players. Although previous studies [13,14] have analysed the percentage of external loads covered during the pre-competition warm-up relative to the total match load, this is the first study to directly compare external load demands between the pre-competition warm-up and official matches, distinguishing by specific playing positions.

The results indicate that official match demands are greater than those of the pre-competition warm-up across all distance-related variables, except for DZ1. Regarding specific positions, higher relative external load values were recorded during official matches compared to the pre-competition warm-up across all positions for TD, DZ2, DZ3, DZ4, and DZ5. However, for DZ1, midfielders covered more distance during competitions, while full-backs and wingers covered more during the pre-competition warm-up. Additionally, midfielders, wingers, and forwards performed a higher number of decelerations (DECs) during competitions, whereas central defenders showed higher DEC values during the pre-competition warm-up. These positional results are largely as expected based on established physical profiles in football. Positions such as midfielders, wingers, and forwards typically engage in more frequent high-intensity efforts and decelerations during match play, which explains their higher values in competition. Conversely, the observed pattern in DZ1 and the decelerations by central defenders during the warm-up may reflect their specific movement patterns and roles within pre-match routines.

To maximise the positive effects of the pre-competition warm-up (e.g., performance enhancement and injury risk reduction), it is crucial to refine the design of this strategy [1]. The warm-up should partially reflect the demands of the competition while carefully adjusting the external load to avoid negatively impacting subsequent performance and to ensure optimal preparation [9].

In this study, when all players were analysed collectively, the demands of official matches were higher than those of the pre-competition warm-up across all distance-related variables, except for DZ1. The percentage differences ranged between 2% and 26%. These findings partially align with previous research by Casamichana et al. [14] and Williams et al. [13], who also demonstrated that external loads during official matches exceeded that of the pre-competition warm-up, although with lower similarity values than those reported in this study. This discrepancy may be attributed to the fact that the aforementioned studies considered absolute load values (i.e., metres), whereas this study focused on relative load values (m/min). These results are generally expected, as official matches typically involve higher intensity demands than warm-ups. However, by using relative values (m/min), our study offers a more accurate reflection of intensity, which may explain the slightly higher similarity values observed. This approach is particularly relevant for practitioners seeking to ensure that warm-up activities adequately stimulate the physiological systems involved in match performance.

The observed close alignment between competitions and warm-ups in high-speed variables (i.e., DZ4 and DZ5, with differences near 2%) is particularly noteworthy. Preparing players for such actions is essential, as these are often among the first to occur in matches, typically performed at maximum intensity. The first 15 min of official matches are generally the most demanding period [21], as shown by time-segmented analyses of match intensity and peak load occurrences. Therefore, it is reasonable to suggest that including high-speed efforts toward the end of the warm-up may serve as an effective means of preparing players for early high-intensity phases of play. The relatively small discrepancy observed in these zones suggests that the current warm-up protocol may already incorporate an adequate volume of sprinting actions; however, further fine-tuning may yield additional performance benefits [22].

Regarding the number of accelerations (ACCs), no significant differences were observed, whereas higher DEC values were recorded during official matches. This could be explained by the muscle damage associated with decelerations, which should be progressively incorporated into the pre-competition warm-up, thus accounting for the minimal differences (i.e., 0.14%). Decelerations impose high eccentric loads on the musculoskeletal system and are crucial for match-specific actions, such as sudden stops, changes of direction, and defensive adjustments. Their inclusion in warm-up activities may also contribute to neuromuscular priming and enhanced proprioceptive readiness, both of which are important for injury prevention [23].

These findings provide valuable insights for adjusting the pre-competition warm-up to align with match demands. Furthermore, understanding warm-up demands allows for more precise weekly load management for each player [14]. Monitoring and modifying the balance between warm-up and match intensities may help practitioners avoid excessive fatigue accumulation, ensure progressive activation of relevant muscle groups, and optimize players’ readiness both physically and mentally [24].

Given the differences observed in external load demands by playing positions in football [15,16], it is essential to compare the relative external load demands (m/min) of the pre-competition warm-up and official matches on an individualised, position-specific basis. This need has been previously highlighted as a future research direction by Casamichana et al. [14]. Positional demands vary considerably in terms of distance covered, speed thresholds reached, and the frequency of high-intensity actions. As such, a universal warm-up protocol for all players might be insufficient or even counterproductive, especially if it fails to activate position-specific movement patterns or energy systems [25].

In this study, higher relative external load values were recorded during official matches compared to the pre-competition warm-up across all positions for TD, DZ2, DZ3, DZ4, and DZ5. However, for DZ1, midfielders covered more distance during competitions, while full-backs and wingers covered more during the pre-competition warm-up. These subtle differences underscore the necessity of tailoring the pre-competition warm-up to the specific demands of each position. A generalised approach for all players may lead to suboptimal results, potentially causing excessive fatigue, which could impair performance and increase the risk of injury [1,22]. Individualisation could involve modifying the volume, intensity, and content of warm-up activities to reflect the dominant actions associated with each position—for example, greater sprint work and decelerations for wide players, or increased change-of-direction drills for midfielders.

Additionally, midfielders, wingers, and forwards performed a higher number of DECs during competition, whereas central defenders showed higher DEC values during the pre-competition warm-up. These findings reinforce the importance of individualising not only training processes but also the pre-competition warm-up, as well as understanding its specific demands for effective load management during the microcycle. For example, coaches may consider progressively integrating eccentric-focused exercises for positions more exposed to high volumes of decelerations, especially when designing warm-up sequences for congested fixture periods.

While this article provides valuable information for football strength and conditioning coaches, it has certain limitations that should be acknowledged. The primary limitation of this study is the absence of internal load metrics (e.g., heart rate, perceived exertion, or blood lactate), which would have provided a more comprehensive picture of the players’ physiological responses to both the warm-up and the official match. This choice was primarily due to logistical constraints during data collection and the observational nature of the study, which was conducted during the regular competitive season of a semi-professional team. Our primary objective was to establish a foundational comparison of external load demands under real-world conditions, while minimizing disruptions to team routines. Nevertheless, we fully acknowledge the value of integrating internal and external load data, and we have highlighted this as a key consideration for future research aimed at enhancing the ecological validity and interpretative depth of similar studies. The second limitation is that only male players were included, potentially limiting the applicability of the findings to female populations. Finally, as a case study, data were collected from a single team (19 players), meaning the results may be influenced by the specific characteristics of the players within that team.

Specific practical recommendations can be derived from the current findings. First, coaches and strength and conditioning professionals should evaluate the degree to which their existing warm-up protocols replicate the intensity and positional demands of competitions. This includes revisiting the volume and timing of high-speed runs and decelerations within the warm-up, especially for positions that rely heavily on such actions. Incorporating sport-specific drills that mimic positional movements and transition phases observed during matches may also enhance performance preparedness.

Second, the design of warm-ups should consider not only physical readiness but also neuromuscular and cognitive activation. Adding decision-making elements to late-stage warm-up activities—for example, reactive sprint tasks or small-sided games with dynamic constraints—may further align warm-up content with match situations [26].

Third, warm-up data should be integrated into the weekly load monitoring systems used by technical staff. This would allow practitioners to contextualise training and competition loads with respect to player readiness, reducing the risk of under- or over-preparation. A mismatch between warm-up and match demands, especially in high-speed or eccentric-load actions, should be addressed proactively during the microcycle.

Finally, future researchers are encouraged to explore how individualised warm-up protocols influence acute match performance and injury risk longitudinally. Including both internal and external load measures and assessing female and youth athletes would broaden the scope and applicability of future evidence. Additionally, the integration of psychological and perceptual metrics could offer a more holistic view of pre-match preparedness and help identify individual responses to different warm-up strategies.

## 5. Conclusions

The demands of official competitions were higher than those of the pre-competition warm-up across all distance-related variables, except for DZ1. Regarding specific positions, relative external load values were greater during official matches compared to the pre-competition warm-up for all positions in terms of TD, DZ2, DZ3, DZ4, and DZ5. However, for DZ1, midfielders covered more distance during competitions, while full-backs and wingers covered more during the pre-competition warm-up.

Additionally, midfielders, wingers, and forwards performed a higher number of decelerations during competitions, whereas central defenders showed higher values during the pre-competition warm-up. No significant differences were observed concerning ACCs

As a practical application of these findings, it is recommended to tailor the pre-competition warm-up according to the specific demands of competitions for each playing position and to incorporate the specific demands of the warm-up into the load dynamics of the microcycle.

## Figures and Tables

**Table 1:** Pre-competitive warm-up description.

Phase	Description	Duration
General mobility	Free movement around the space, performing general warm-up exercises (high knees, heel flicks, hip abduction and adduction, lateral runs, jumps, etc.).	1′ 30″
Group-specific mobility	In a circle, guided mobility, dynamic stretching, and basic strength exercises for activation.	1′ 30″
Dynamic mobility in lines	Guided mobility in two 10 m lines (skipping, heel flicks, lateral runs, hip abduction and adduction, lateral crossover runs, jumps, stride length exercises, direction changes, and accelerations). Includes four 20 m sprints after ladder drills.	3′
Hydration break (1′)		
Ball work in pairs	With one ball per pair, players standing 3 metres apart perform specific match-related technical actions (heading, control, and passing; dribbling; and feinting) and free long passing.	3′
Small-sided game (4 vs. 4 + 2c)	Possession-based game (unlimited touches, except for the two neutral players, who are limited to two touches) in a 20 × 15 m space. Teams are arranged by field relationships + 2 neutral players (midfielders).	2 × 2′; 30″ rest
Hydration break (1′)		
Specific attacking actions	The team performs different finishing drills: (1) The fitness coach plays the ball to the striker, who controls and shoots on goal. (2) Ball circulation followed by a cross into the box, with two strikers and the opposite winger or full-back attempting to finish. (3) The fitness coach introduces a third ball for the midfielder who did not start the circulation to shoot from outside the box.	3′ 30″
Speed drills	Four 5 m sprints are performed after completing a preceding exercise (high knees, jumping headers, direction changes, and backwards direction changes).	1′

**Table 2:** Differences in external load demands between pre-competitive warm-up and official matches considering the whole team (n = 225 observations).

Variable	Warm Up Mean ± SD	Game Mean ± SD	Δ (%)	*p*	ES
TD (m/min)	80.73 ± 8.12	107.12 ± 13.48	−26.39	<0.001	−2.03
DZ1 (m/min)	67.06 ± 8.91	66.40 ± 8.61	0.65	0.198	0.22
DZ2 (m/min)	11.00 ± 5.95	28.41 ± 7.65	−17.42	<0.001	−2.17
DZ3 (m/min)	1.83 ± 1.29	6.72 ± 2.79	−4.89	<0.001	−1.91
DZ4 (m/min)	0.70 ± 0.51	3.32 ± 1.24	−2.62	<0.001	−1.86
DZ5 (m/min)	0.15 ± 0.25	2.27 ± 1.32	−2.12	<0.001	−1.39
ACC (nº/min)	0.97 ± 0.32	0.98 ± 0.21	−0.01	0.579	0.09
DEC (nº/min)	0.95 ± 0.33	1.09 ± 0.23	−0.14	<0.001	−0.28

Note: SD = standard deviation; Δ (%): percentage change between warm-up and match; *p* = significance level; ES = effect size; TD = total distance; DZ1 = distance travelled from 0 to 12 km·h^−1^; DZ2 = from 12.1 to 18 km·h^−1^; DZ3 = from 18.1 to 21 km·h^−1^; DZ4 = from 21.1 to 24 km·h^−1^; DZ5 = over 24 km·h^−1^; ACC = number of accelerations above 3 m·s^−2^; DEC = decelerations above −3 m·s^−2^.

**Table 3:** Differences in external load demands between pre-competitive warm-up and official matches in central defenders (n = 45 observations).

Variable	Warm Up Mean ± SD	Game Mean ± SD	Δ (%)	*p*	ES
TD (m/min)	77.88 ± 4.88	98.74 ± 7.78	−20.86	<0.001	−2.13
DZ1 (m/min)	63.99 ± 14.61	64.84 ± 14.99	−0.84	0.387	−0.13
DZ2 (m/min)	11.27 ± 11.91	24.33 ± 8.15	−13.06	<0.001	−2.32
DZ3 (m/min)	1.84 ± 2.29	5.82 ± 4.74	−3.97	<0.001	−1.36
DZ4 (m/min)	0.66± 0.46	2.33 ± 1.04	−1.67	<0.001	−1.84
DZ5 (m/min)	0.12 ± 0.23	1.42 ± 0.77	−1.31	<0.001	−1.67
ACC (nº/min)	1.04 ± 0.21	0.97 ± 0.16	0.07	0.052	0.30
DEC (nº/min)	1.14 ± 0.24	1.00 ± 0.14	0.13	<0.001	0.58

Note: SD = standard deviation; Δ (%): percentage change between warm-up and match; *p* = significance level; ES = effect size; TD = total distance; DZ1 = distance travelled from 0 to 12 km·h^−1^; DZ2 = from 12.1 to 18 km·h^−1^; DZ3 = from 18.1 to 21 km·h^−1^; DZ4 = from 21.1 to 24 km·h^−1^; DZ5 = over 24 km·h^−1^; ACC = number of accelerations above 3 m·s^−2^; DEC = decelerations above −3 m·s^−2^.

**Table 4:** Differences in external load demands between pre-competitive warm-up and official matches in full-backs (n = 55 observations).

Variable	Warm Up Mean ± SD	Game Mean ± SD	Δ (%)	*p*	ES
TD (m/min)	82.36 ± 6.76	104.33 ± 13.42	−21.97	<0.001	−1.91
DZ1 (m/min)	69.01 ± 5.22	66.86 ± 4.64	2.14	0.009	0.36
DZ2 (m/min)	10.68 ± 2.32	25.42 ± 6.65	−14.74	<0.001	−2.45
DZ3 (m/min)	1.69 ± 0.57	6.09 ± 2.03	−4.41	<0.001	−2.09
DZ4 (m/min)	0.76 ± 0.47	3.40 ± 1.31	−2.65	<0.001	−1.76
DZ5 (m/min)	0.24 ± 0.31	2.55 ± 1.19	−2.32	<0.001	−1.73
ACC (nº/min)	0.98 ± 0.35	1.02 ± 0.24	−0.03	0.516	−0.09
DEC (nº/min)	1.06 ± 0.38	1.13 ± 0.28	−0.07	0.180	−0.18

Note: SD = standard deviation; Δ (%): percentage change between warm-up and match; *p* = significance level; ES = effect size; TD = total distance; DZ1 = distance travelled from 0 to 12 km·h^−1^; DZ2 = from 12.1 to 18 km·h^−1^; DZ3 = from 18.1 to 21 km·h^−1^; DZ4 = from 21.1 to 24 km·h^−1^; DZ5 = over 24 km·h^−1^; ACC = number of accelerations above 3 m·s^−2^; DEC = decelerations above −3 m·s^−2^.

**Table 5:** Differences in external load demands between pre-competitive warm-up and official matches in midfielders (n = 47 observations).

Variable	Warm Up Mean ± SD	Game Mean ± SD	Δ (%)	*p*	ES
TD (m/min)	78.68 ± 7.11	112.69 ± 9.35	−34.01	<0.001	−3.25
DZ1 (m/min)	65.04 ± 5.81	67.62 ± 4.94	−2.58	0.033	−0.32
DZ2 (m/min)	10.77 ± 3.15	33.21 ± 4.87	−22.44	<0.001	−4.60
DZ3 (m/min)	2.16 ± 1.32	7.09 ± 1.62	−4.93	<0.001	−2.46
DZ4 (m/min)	0.62 ± 0.57	3.16 ± 0.83	−2.54	<0.001	−2.59
DZ5 (m/min)	0.10 ± 0.23	1.62 ± 0.87	−1.52	<0.001	−1.69
ACC (nº/min)	0.90 ± 0.38	0.98 ± 0.22	−0.08	0.128	−0.23
DEC (nº/min)	0.70 ± 0.24	1.04 ± 0.19	−0.34	<0.001	−1.22

Note: SD = standard deviation; Δ (%): percentage change between warm-up and match; *p* = significance level; ES = effect size; TD = total distance; DZ1 = distance travelled from 0 to 12 km·h^−1^; DZ2 = from 12.1 to 18 km·h^−1^; DZ3 = from 18.1 to 21 km·h^−1^; DZ4 = from 21.1 to 24 km·h^−1^; DZ5 = over 24 km·h^−1^; ACC = number of accelerations above 3 m·s^−2^; DEC = decelerations above −3 m·s^−2^.

**Table 6:** Differences in external load demands between pre-competitive warm-up and official matches in wingers (n = 40 observations).

Variable	Warm Up Mean ± SD	Game Mean ± SD	Δ (%)	*p*	ES
TD (m/min)	82.76 ± 12.77	105.21 ± 15.22	−22.45	<0.001	−1.87
DZ1 (m/min)	69.09 ± 9.42	65.72 ± 8.21	−0.08	0.014	0.41
DZ2 (m/min)	11.39 ± 3.95	25.82 ± 5.59	−0.24	<0.001	−2.92
DZ3 (m/min)	1.55 ± 0.67	6.70 ± 1.65	−3.37	<0.001	−3.35
DZ4 (m/min)	0.61 ± 0.47	3.72 ± 0.91	−14.42	<0.001	−3.24
DZ5 (m/min)	0.12 ± 0.21	3.24 ± 1.32	−5.15	<0.001	−2.41
ACC (nº/min)	0.95 ± 0.29	1.02 ± 0.19	−3.12	0.087	−0.28
DEC (nº/min)	0.89 ± 0.31	1.12 ± 0.24	−3.13	<0.001	−0.70

Note: SD = standard deviation; Δ (%): percentage change between warm-up and match; *p* = significance level; ES = effect size; TD = total distance; DZ1 = distance travelled from 0 to 12 km·h^−1^; DZ2 = from 12.1 to 18 km·h^−1^; DZ3 = from 18.1 to 21 km·h^−1^; DZ4 = from 21.1 to 24 km·h^−1^; DZ5 = over 24 km·h^−1^; ACC = number of accelerations above 3 m·s^−2^; DEC = decelerations above −3 m·s^−2^.

**Table 7:** Differences in external load demands between pre-competitive warm-up and official matches in forwards (n = 38 observations).

Variable	Warm Up Mean ± SD	Game Mean ± SD	Δ (%)	*p*	ES
TD (m/min)	82.16 ± 6.61	116.21 ± 13.67	−34.05	<0.001	−2.35
DZ1 (m/min)	68.21 ± 4.85	66.81 ± 6.57	1.40	0.300	0.17
DZ2 (m/min)	11.03 ± 2.92	34.40 ± 6.44	−23.37	<0.001	−3.25
DZ3 (m/min)	1.90 ± 0.70	8.24 ± 2.02	−6.35	<0.001	−3.38
DZ4 (m/min)	0.88 ± 0.55	4.13 ± 1.30	−3.26	<0.001	−2.96
DZ5 (m/min)	0.14 ± 0.24	2.62 ± 1.52	−2.48	<0.001	−1.70
ACC (nº/min)	0.99 ± 0.31	0.92 ± 0.20	0.08	0.074	0.30
DEC (nº/min)	0.94 ± 0.27	1.18 ± 0.24	−0.23	<0.001	−0.87

Note: SD = standard deviation; Δ (%): percentage change between warm-up and match; *p* = significance level; ES = effect size; TD = total distance; DZ1 = distance travelled from 0 to 12 km·h^−1^; DZ2 = from 12.1 to 18 km·h^−1^; DZ3 = from 18.1 to 21 km·h^−1^; DZ4 = from 21.1 to 24 km·h^−1^; DZ5 = distance travelled over 24 km·h^−1^; ACC = number of accelerations above 3 m·s^−2^; DEC = decelerations above −3 m·s^−2^.

## Data Availability

Data supporting the findings of this study are available from the corresponding author upon reasonable request.

## References

[1] Hills S.P., Barrett S., Hobbs M., Barwood M.J., Radcliffe J.N., Cooke C.B., Russell M. (2020). Modifying the Pre-Pitch Entry Practices of Professional Soccer Substitutes May Contribute towards Improved Movement-Related Performance Indicators on Match-Day: A Case Study. PLoS ONE.

[2] Soligard T., Myklebust G., Steffen K., Holme I., Silvers H., Bizzini M., Junge A., Dvorak J., Bahr R., Andersen T.E. (2008). Comprehensive warm-up programme to prevent injuries in young female footballers: Cluster randomised controlled trial. BMJ.

[3] Bishop D. (2003). Warm up I: Potential Mechanisms and the Effects of Passive Warm up on Exercise Performance. Sports Med..

[4] Bishop D. (2003). Warm up II: Performance Changes Following Active Warm up and How to Structure the Warm Up. Sports Med..

[5] Gregson W.A., Batterham A., Drust B., Cable N.T. (2005). The Influence of Pre-Warming on the Physiological Responses to Prolonged Intermittent Exercise. J. Sports Sci..

[6] Filter A., Olivares-Jabalera J., Dos’Santos T., Madruga M., Lozano J., Molina A., Santalla A., Requena B., Loturco I. (2023). High-Intensity Actions in Elite Soccer: Current Status and Future Perspectives. Int. J. Sports Med..

[7] Faude O., Koch T., Meyer T. (2012). Straight Sprinting Is the Most Frequent Action in Goal Situations in Professional Football. J. Sports Sci..

[8] Hammami A., Zois J., Slimani M., Russel M., Bouhlel E. (2018). The Efficacy and Characteristics of Warm-up and Re-Warm-up Practices in Soccer Players: A Systematic Review. J. Sports Med. Phys. Fit..

[9] Ben Brahim M., Sal-De-Rellán A., García-Valverde A., Yasin H., Raya-González J. (2023). The Effect of Three Different Pre-Match Warm-up Structures on Male Professional Soccer Players’ Physical Fitness. PeerJ.

[10] Taylor J.M., Weston M., Portas M.D. (2013). The Effect of a Short Practical Warm-up Protocol on Repeated Sprint Performance. J. Strength Cond. Res..

[11] van den Tillaar R., von Heimburg E. (2016). Comparison of Two Types of Warm-Up Upon Repeated-Sprint Performance in Experienced Soccer Players. J. Strength Cond. Res..

[12] Patti A., Giustino V., Hirose N., Messina G., Cataldi S., Grigoli G., Marchese A., Mulè G., Drid P., Palma A. (2022). Effects of an Experimental Short-Time High-Intensity Warm-up on Explosive Muscle Strength Performance in Soccer Players: A Pilot Study. Front. Physiol..

[13] Williams J.H., Jaskowak D.J., Williams M.H. (2019). Warm-Up and Match Demands How Much Does the Warm-Up Contribute to the Soccer Match-Day Load?. Sport Perform. Sci. Rep..

[14] Casamichana D., Barba E., Nakamura F.Y., Agirrezabalaga O., Castellano J. (2024). Comparison of External Load during Pre-Match Warm-up among Different Age Categories from the Same Football Professional Club. Kinesiology.

[15] García-Calvo T., Ponce-Bordón J.C., Pons E., del Campo R.L., Resta R., Raya-González J. (2022). High Metabolic Load Distance in Professional Soccer According to Competitive Level and Playing Positions. PeerJ.

[16] Lobo-Triviño D., García-Calvo T., Rubio-Morales A., Nevado F., Chena M., Piñero-Madrona J.A., Martín-Ardila E., Raya-González J. (2024). The Influence of Match Status on the Conditional Characteristics of Tactical Sprint Actions in Professional Soccer Players. Biol. Sport.

[17] McKay A.K.A., Stellingwerff T., Smith E.S., Martin D.T., Mujika I., Goosey-Tolfrey V.L., Sheppard J., Burke L.M. (2022). Defining Training and Performance Caliber: A Participant Classification Framework. Int. J. Sports Physiol. Perform..

[18] Scott M.T.U., Scott T.J., Kelly V.G. (2016). The Validity and Reliability of Global Positioning Systems in Team Sport: A Brief Review. J. Strength Cond. Res..

[19] Cormier P., Tsai M.C., Meylan C., Agar-Newman D., Epp-Stobbe A., Kalthoff Z., Klimstra M. (2023). Concurrent Validity and Reliability of Different Technologies for Sprint-Derived Horizontal Force-Velocity-Power Profiling. J. Strength Cond. Res..

[20] Cohen J. (1988). Statistical Power Analysis for the Behavioural Sciences.

[21] DiMascio M., Bradley P.S. (2013). Evaluation of the Most Intense High-Intensity Running Period in English FA Premier League Soccer Matches. J. Strength Cond. Res..

[22] Buchheit M., Simpson B.M. (2017). Player-Tracking Technology: Half-Full or Half-Empty Glass?. Int. J. Sports Physiol. Perform..

[23] Dalen T., Ingebrigtsen J., Ettema G., Hjelde G.H., Wisløff U. (2016). Player Load, Acceleration, and Deceleration During Forty-Five Competitive Matches of Elite Soccer. J. Strength Cond. Res..

[24] Thorpe R.T., Strudwick A.J., Buchheit M., Atkinson G., Drust B., Gregson W. (2015). Monitoring Fatigue During the In-Season Competitive Phase in Elite Soccer Players. Int. J. Sports Physiol. Perform..

[25] Mara J.K., Thompson K.G., Pumpa K.L., Morgan S. (2017). The Acceleration and Deceleration Profiles of Elite Female Soccer Players During Competitive Matches. J. Sci. Med. Sport.

[26] Giuriato M., Lovecchio N. (2018). Cognitive Training in Soccer: Where Is the Key Point?. Open Access Libr. J..

